# The Week's Work

**Published:** 1910-11-19

**Authors:** 


### The Week's Work.

### OPERATIONS IN CHILDREN'S WARDS.

If we were simply to state that it was the invariable practice in a hospital to operate upon children in the children's wards without any screen or privacy, we should properly be met with remonstrance and repudiation by every humane and competent hospital manager. To say this is to define clearly that it is indefensible and wrong to operate in this way on the patients in children's wards of any institution. According to a report in the *Eastern Morning News* and other papers, it appears to be the practice at the Hull (Fever) Sanatorium to operate on the children in the children's wards of that institution, cases of tracheotomy so treated having formed the subject of a fairly animated discussion at recent meetings of the Hull Corporation Hospital Committee. A practice like this is only on a par with that which prevailed at a Wolverhampton hospital, which we commented on some months ago, where the operation theatre contained two beds in addition to the operation table to enable operations to be conducted rapidly, but in the presence of other patients. From the report in the newspapers it would appear that complaints of operations in children's wards at Hull have been made in the public Press. Dr. Robinson described the procedure of such an operation at Hull upon a child in the children's ward in full view of all the little inmates. He is reported to have said: "Nurses were bustling about making preparations for the operation. The children could see, and became alarmed. They knew that the child in the advanced stages of diphtheria could not scream, but it struggled and kicked whilst the chloroform was being administered, and it was not right that this should be done in the ward where other children were." We entirely agree with the humane remarks of Dr. Robinson, which do not appear to have been approved, however, by the majority of the members of the Committee then present, for they passed a resolution to refer the matter of providing an operation theatre back to the medical officer and architect, the latter of whom had reported what amounted to insurmountable difficulties which prevented the provision of a convenient theatre without great cost. As matters stand at present, the children in the Hull Sanatorium who may need an operation and their associates in the children's wards are, it would appear, to suffer grave wrong at the hands of the public authorities responsible for the management of this institution for want of a little expert advice, brains, and some reasonable generosity in the supply of funds. We shall wait with interest to see whether the citizens of Hull, a great city, will permit the present state of affairs in regard to these children in their care to go on unchanged, or whether public opinion will promptly step in and compel, if necessary, those responsible to do their duty without a moment's avoidable delay. We wish that Mr. John Burns would appoint two expert commissioners, outside the Government service altogether, to make an independent inspection, and report upon every fever hospital, and all provision for infectious cases throughout England. We believe if this were done the results would not only be an immense protection to thousands of poor people and children, but that many abuses would thus probably be brought to light which might amaze the general public, who mostly think of a fever hospital with dislike or terror.

### NORTH STAFFORDSHIRE INFIRMARY.

On October 2, 1909, we published a report by Sir Henry Burdett urging the immediate overhauling of the North Staffordshire Infirmary, Hartshill, in respect to its building and departments, and also in regard to its constitution and rules. That report exhaustively dealt with the defects and requirements of this infirmary in all departments under both heads. Thanks largely to the energy and devotion of Mr. W. D. Spanton, F.R.C.S., and the members of the Reconstruction Committee, we were able to report on June 25 last that the North Staffordshire Infirmary buildings will in due course be brought up to date, and that this hospital would so attain to the modern standard. To-day we have the pleasure of reporting that at the meeting of the governors held on the 8th instant the revised rules and regulations were submitted and approved, practically without amendment, a fact which testifies to the thoroughness with which the Committee on Bye-laws has discharged the important duty entrusted to it.

### ITS COMPLETE REORGANISATION.

The bye-laws as they stand are a great improvement on the old ones. In the matter of representation the new rules provide fairly and liberally for the representation of establishment governors and Hospital Saturday governors as representatives of the artisans who collectively contribute each year upwards of 60 per cent. of the annual ordinary income of this hospital. An attempt was made to reduce the representation of the working classes, but was wisely defeated by the governors, who properly held that it is of the first importance to encourage the adequate representation of all classes, and especially of the working classes, in the case of a hospital like that at Stoke, which depends so largely upon this source of revenue for its efficiency and maintenance. The bye-laws further provide for an increase in the staff by the selection of one extra honorary physician, honorary surgeon, assistant physician, and assistant surgeon in addition to other special appointments. We hope that the General Committee will promptly take steps to advertise these new appointments, and that every care will be taken to select for them whichever of the candidates may offer best evidence that their qualifications, experience, and abilities are in fact the highest. We observe that, no doubt through an oversight, having regard to the size and importance of the North Staffordshire Infirmary, although provision is made for the appointment of an assistant secretary, there is no provision under the rules for the appointment of an assistant matron or lady superintendent. Having regard to the serious character of the majority of the cases treated in this hospital, and to the onerous work entailed upon the matron in regard to the nursing staff, we hope that this oversight in the rules will be speedily rectified by moving the governors to give authority for the appointment of an assistant matron, whose services are essential, if the highest efficiency and full justice to the resident staff are, as we have no doubt, the aims which every manager is anxious to secure. We congratulate all concerned upon the spirit in which they have taken the drastic criticisms contained in Sir Henry Burdett's report, and on the thoroughness with which they have set themselves to put everything connected with the North Staffordshire Infirmary in as perfect order as possible. It is only just that we should express a warm meed of thanks to the local press, and especially to the *Staffordshire Sentinel*, for the whole-hearted and continuous support which they have extended to the party of reform and thereby helped to secure that every recommendation made by Sir Henry Burdett has been completely and satisfactorily carried out.

### THE CHARING CROSS HOSPITAL APPEAL.

Charing Cross Hospital this week issues a formal appeal for the sum of £100,000 which it wishes to raise in order to free itself from debt. The letter of the President of the Appeal Committee has been given wide publicity, and is worthy of serious attention by those who are interested in metropolitan hospital work. Charing Cross Hospital occupies a position which is somewhat unique among London hospitals. It caters not merely for its own immediate hospital population, but for a very wide circle outside the radius of its own particular "sphere of influence." As the letter of the President succinctly points out, from its very situation the hospital is pre-eminently an accident hospital. "Cases are brought in from all parts of the busy neighbourhood around—from business houses, hotels, clubs, restaurants, factories, railway stations, and from the streets themselves, so increasingly dangerous since the advent of motor traffic. Moreover, the adjacent thoroughfares are highways for all sorts and conditions of people from all over the world, and the hospital is called upon to perform not merely a local but a universal service." These points should all be carefully remembered in estimating the desirability of responding to the appeal. And there can be no doubt that the hospital stands in need of support. The urgent need of the institution arises from the fact that it has a large mortgage debt, and the annual payments for interest and sinking fund in respect of this debt are a tremendous drain upon its income. In consequence, the Council has been forced to close several wards in order to reduce expenditure. Five wards, containing eighty-seven beds, are now closed, which means that some fifteen hundred sick persons are deprived of the benefits of this charity every year. These closed wards and beds could all be filled, for the waiting list of the hospital has always from eighty to a hundred names upon it, and the less serious of these cases have to wait for weeks, and sometimes months, before being admitted. Even casualties that ought to be taken in have frequently to be sent on to other hospitals, or to the infirmary, because the beds are full. The out-patient department has recently been much improved and enlarged, and the hospital is thoroughly efficient and up-to-date. What is wanted is money to enable it to utilise its beds and thereby to render it, what it might easily be, one of the most useful of metropolitan hospitals. We cordially commend the appeal to the interest of all hospital workers.

### THE DRUG-ROOM BEETLE.

In a paper read at an evening meeting of the Pharmaceutical Society, Professor H. G. Greenish and Miss D. M. Braithwaite described a method by which it can be shown whether powdered drugs are prepared from worm-eaten or fresh samples. The insect which infests drugs is a small brown beetle, from 2 to 3 mm. in length, and is known as the drug-room beetle, *Sitodrepa panicea*, but when present in powdered drugs the proportion is usually so small that it cannot be detected by direct examination under the microscope. The authors have, however, devised a method by which this difficulty can be overcome and the presence of the merest traces detected, namely, by destroying all the organic matter of the powdered drug and separating the particles of beetle, which can then be readily observed under the microscope. The beetle resists chemical reagents of all kinds, and this property renders the process of separation possible. The importance of this paper lies in the fact that by no other means is it possible to show whether a powdered drug has been prepared from drugs that have been attacked by worms, so that in future greater care should be used in selecting drugs for powdering.

### THIS WEEK'S DRUG MARKET.

The demand for drugs which are usually in request during the winter season has set in, but otherwise business is not very brisk. Cod-liver oil is tending slightly higher in value, and the present prices are probably as low as any that are likely to be ruling during the next month or two. Ipecacuanha, after receding in value to some extent, has advanced again, and it seems probable that further advances may take place. Eucalyptus oil is in good request, but prices are unchanged. As yet there has been no further advance in the price of glycerine, but refiners are expected to raise the value any day. Oil of cloves is again dearer as a result of further advances in the value of cloves. Saffron is dearer. Ergot of rye continues scarce, and prices are rising. Opium is in rather better demand, and the tendency is to slightly higher values. Indian hemp, the price of which has for a long time been stationary, has this week been sold at slightly dearer rates. Liquid extract of male fern is tending cheaper. It is anticipated that the prices of tannic and gallic acids will be raised. Cream of tartar and tartaric acid are not appreciably higher in price since last week.

